# BIRC5 expression by race, age and clinical factors in breast cancer patients

**DOI:** 10.1186/s13058-024-01792-y

**Published:** 2024-03-21

**Authors:** Alina M. Hamilton, Andrea Walens, Sarah C. Van Alsten, Linnea T. Olsson, Joseph Nsonwu-Farley, Xiaohua Gao, Erin L. Kirk, Charles M. Perou, Lisa A. Carey, Melissa A. Troester, Yara Abdou

**Affiliations:** 1 Lineberger Comprehensive Cancer Center, University of North Carolina, Chapel Hill, NC 27599 USA; 2 Department of Pathology and Laboratory Medicine, School of Medicine, University of North Carolina at Chapel Hill, Chapel Hill, NC 27599 USA; 3 Department of Epidemiology, Gillings School of Public Health, University of North Carolina at Chapel Hill, Chapel Hill, NC 27599 USA; 4 Department of Genetics, Lineberger Comprehensive Cancer Center, University of North Carolina, Chapel Hill, NC 27599 USA; 5 Department of Medicine, Division of Oncology, University of North Carolina at Chapel Hill, 101 Manning Drive, CB# 7305, Chapel Hill, NC 27514 USA

## Abstract

**Purpose:**

Survivin/BIRC5 is a proliferation marker that is associated with poor prognosis in breast cancer and an attractive therapeutic target. However, BIRC5 has not been well studied among racially diverse populations where aggressive breast cancers are prevalent.

**Experimental design:**

We studied BIRC5 expression in association with clinical and demographic variables and as a predictor of recurrence in 2174 participants in the Carolina Breast Cancer Study (CBCS), a population-based study that oversampled Black (n = 1113) and younger (< 50 years; n = 1137) participants with breast cancer. For comparison, similar analyses were conducted in The Cancer Genome Atlas [TCGA N = 1094, Black (n = 183), younger (n = 295)]. BIRC5 was evaluated as a continuous and categorical variable (highest quartile vs. lower three quartiles).

**Results:**

Univariate, continuous BIRC5 expression was higher in breast tumors from Black women relative to non-Black women in both estrogen receptor (ER)-positive and ER-negative tumors and in analyses stratified by stage (i.e., within Stage I, Stage II, and Stage III/IV tumors). Within CBCS and TCGA, BIRC5-high was associated with young age (< 50 years) and Black race, as well as hormone receptor-negative tumors, non-Luminal A PAM50 subtypes, advanced stage, and larger tumors (> 2 cm). Relative to BIRC5-low, BIRC5-high tumors were associated with poor 5-year recurrence-free survival (RFS) among ER-positive tumors, both in unadjusted models [HR (95% CI): 2.7 (1.6, 4.6)] and after adjustment for age and stage [Adjusted HR (95% CI): 1.87 (1.07, 3.25)]. However, this relationship was not observed among ER-negative tumors [Crude HR (95% CI): 0.7 (0.39, 1.2); Adjusted HR (95% CI): 0.67 (0.37, 1.2)].

**Conclusion:**

Black and younger women with breast cancer have a higher burden of BIRC5-high tumors than older and non-Black women. Emerging anti-survivin treatment strategies may be an important future direction for equitable breast cancer outcomes.

**Supplementary Information:**

The online version contains supplementary material available at 10.1186/s13058-024-01792-y.

## Introduction

Black women suffer 41% higher breast cancer mortality compared to White women [1]. Differences in tumor biology at diagnosis (either due to differential risk factors or differences in detection) may contribute to this underlying disparity [2–8]. While research and treatment advances have significantly lowered breast cancer mortality rates over the years, declines in mortality among Black women continue to lag behind [1]. Therefore, there is a vital need for novel targets for therapeutic response in diverse breast cancer patients. Survivin is a protein in the inhibitor of apoptosis protein family encoded by the BIRC5 gene, and its mechanisms of action include inhibition of apoptosis, dysregulation of mitosis, cell cycle progression, carcinogenesis, and DNA repair [9].

Survivin is a marker of poor prognosis [10, 11] and is commonly associated with enhanced proliferative index [12], reduced levels of apoptosis [13], resistance to chemotherapy [14–16], and increased rate of tumor recurrence [17] across multiple tumor types, including breast cancer. Survivin/BIRC5 is already included as a proliferation marker in two clinically utilized RNA-based prognostic assays in breast cancer, including the Oncotype DX assay [18] and Prosigna assay [19]. Prior studies have shown that high survivin expression is associated with estrogen receptor (ER)-negative [20, 21], high grade, and lymph node-positive breast tumors [22, 23]. However, most studies investigating survivin have been conducted in smaller cohorts of predominantly White women or that did not report on race [10, 11, 20–24], and little is known about survivin in tumors from young and Black breast cancer patients, who are more frequently diagnosed with advanced disease, higher grade, and aggressive molecular subtypes [25, 26]. Currently, there are various methods of targeting survivin therapeutically, including small molecule inhibitors that block the function of survivin, interference with survivin gene expression, or survivin-based immunotherapy [27], making this a promising candidate for addressing disparities in outcomes.

Given that survivin/BIRC5 may be an attractive target for aggressive and resistant malignancies that lack effective therapies, we evaluated RNA expression of BIRC5 according to clinical and demographic variables in a large and diverse study population, the Carolina Breast Cancer Study (CBCS; N = 2174 cases: 1113 Black and 1061 non-Black; 1137 < 50 and 1037 ≥ 50 years of age) and compared these findings to those in the Cancer Genome Atlas (TCGA; N = 1095 cases: 183 Black and 816 non-Black; 295 < 50 and 798 ≥ 50 years of age). We hypothesized that in a diverse patient population, BIRC5 would be associated with aggressive disease and recurrence, suggesting potential value in targeted therapy.

## Methods

### Study population

The Carolina Breast Cancer Study (CBCS) [28] is a population-based study that utilized rapid case ascertainment with the North Carolina Central Cancer Registry to identify women aged 20–74 years across 44 counties diagnosed with first primary breast cancer. Recruitment occurred in three phases: 1993–1996 (Phase 1), 1996–2001 (Phase 2), and 2008–2013 (Phase 3). Black women and younger women (< 50 years of age) were oversampled using randomized recruitment [28], such that the final study population is 50% Black and 50% < 50 years old at diagnosis. Out of 4806 invasive breast cancer cases enrolled across all phases, 2174 bulk tumor samples were profiled by Nanostring (Phase 1: N = 259; Phase 2: N = 491; Phase 3: N = 1424). Exclusions included samples with depleted tissue (n = 1188, predominantly from CBCS1/2) or samples with low-quality or insufficient RNA (n = 241). This study was approved by the University of North Carolina at Chapel Hill (UNC-CH) School of Medicine Institutional Review Board in accordance with the revised U.S. Common Rule, and participants provided written informed consent.

### Demographic and clinical characteristics

Health history and demographic variables were collected by a nurse during in-home interviews. Race was self-reported and categorized as Black and non-Black; > 94.7% of non-Black participants self-reported as White (n = 1005), while < 5.3% self-identified as either multiracial (n = 9, 0.85%), Hispanic (n = 15, 1.41%), American Indian/Eskimo (n = 8, 0.75%), Asian or Pacific Islander (n = 23, 2.17%) or Arab (n ≤ 5, < 1%). Importantly, we interpret race herein under a cells-to-society framework [29, 30], that defines race as a social construct, representing the culmination of biological, social (individual and community-level), and environmental exposures that differ by self-reported race. Tumor size, AJCC stage, estrogen receptor (ER), progesterone receptor (PR), and HER2 receptor status were abstracted from medical records and pathology reports.

Recurrence data were available for CBCS Phase 3 (2008–2013; n = 1424). Recurrence-free survival (RFS) was defined as the time between the date of diagnosis to the first local, regional, or distant breast cancer recurrence and verified through medical record review. Recurrence data are complete through October 2019, with a 5-year follow-up completed for all study participants. Among 1424 eligible women, 50 participants were stage IV at diagnosis and excluded from the recurrence analysis. Among 1374 patients with Stage I-III breast cancer, 159 recurrences were identified within 5 years.

### Gene expression data

#### Normalization, molecular subtyping, and BIRC5

RNA was isolated from bulk tumor tissue using the Qiagen FFPE RNeasy isolation kit (Germantown, MD), assayed using Nanostring nCounter technology (Seattle, Washington), and normalized using Remove Unwanted Variation (RUV) as previously described [31–33]. PAM50 molecular subtyping was performed using a research version of the predictor to classify tumors as Luminal A, Luminal B, HER2-Enriched, Basal-like, or Normal-like, and to generate proliferation and risk of recurrence scores (ROR-PT) incorporating tumor size, proliferation and subtype [31, 34].

BIRC5 was considered as both a continuous and categorical variable. For continuous measures of BIRC5, log2-transformed gene expression was utilized in all analyses. Standardized clinical cutpoints do not exist for survivin/BIRC5, and while it is a target of both OncotypeDX [18] and Prosigna [19] multi-gene assays, single gene levels are not established. Therefore, for use as a categorical variable, BIRC5 expression was dichotomized into BIRC5-low and BIRC5-high expression categories using the upper limit of the third expression quartile as a cut point (Log2 3rd quartile cutpoint: CBCS = 7.6; TCGA = 9.4). Differences in the expression of BIRC5 between CBCS and TCGA are likely a result of the different mRNA platforms used in each study (i.e., NanoString in CBCS, RNAseq in TCGA). All tumors were treatment naïve at the time of collection and prior to NanoString assay assessing BIRC5 mRNA expression.

### Statistical analysis

Continuous BIRC5 expression levels were compared across race and clinical tumor characteristics using Welch’s two-sample t-tests. Generalized linear models (glm) with binomial distribution and the identity link function were used to calculate relative frequency differences (RFDs) and 95% confidence intervals (CIs) as the measure of association between BIRC5 expression categories and covariates of interest in CBCS. RFDs are defined as the percentage difference between index and referent groups, namely, the relative frequency of BIRC5-high tumors across demographic and clinical variables. With smaller sample sizes, RFDs could not be computed for TCGA because several models failed to converge. Thus, to measure the strength of association between BIRC5-high and covariates of interest in both CBCS and TCGA, multivariate logistic regression was used to calculate odds ratios (ORs) and 95% CIs. Multivariable models were adjusted for age and race according to the CBCS randomized recruitment design in reduced models, and additionally adjusted for ER status and tumor stage in full models. In models comparing age or race, age comparisons were only adjusted for race, and race comparisons were only adjusted for age. Similarly, in models additionally adjusting for ER status and stage, ER comparisons were only adjusted for stage, and stage comparisons were only adjusted for ER status. Multivariable analyses relied on complete case analysis as rates of missingness were < 1.3% for all covariates. Normal-like tumors were excluded from analyses because this subtype arises from insufficient tumor cellularity [31].

Kaplan–Meier curves and log-rank tests were used to compare mean time to recurrence across BIRC5 categories in stage I-III cases (n = 1374). Recurrence analyses were stratified according to clinical breast cancer subtypes (i.e., ER-positive/Her2-, and TNBC) and were performed across all tumor subtypes, overall. Hazard ratios (HR) and 95% CI were calculated using crude and multivariate Cox proportional hazard models adjusted for patient age and tumor stage. The Wald p-value was used to assess the assumption of proportionality. While there was evidence of non-proportional hazards, point estimates did not differ substantially between models. All statistical analyses were performed in R version 4.0.3.

### Data availability

RNA sequencing and clinical data from TCGA breast cancer dataset, including 1095 primary tumors, were used to compare and validate BIRC5 relationships identified in CBCS. These data are publicly available under dbGaP accession phs000178.v1.p1, with additional data available at https://gdc.cancer.gov/about-data/publications/PanCan-CellOfOrigin^35^. CBCS data are available upon request (https://unclineberger.org/cbcs).

## Results

### BIRC5 expression, patient and tumor characteristics

**Fig. 1:**
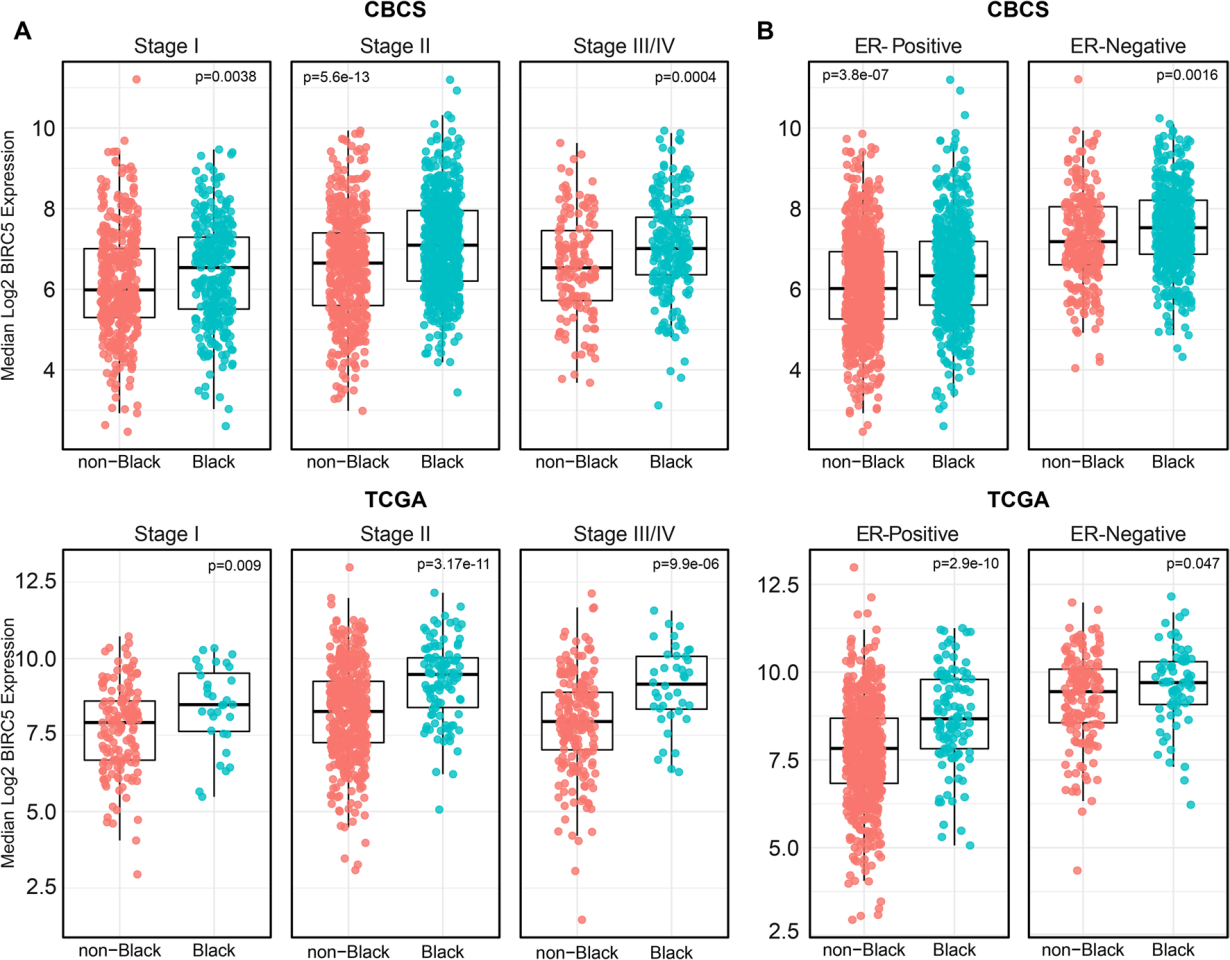
BIRC5 Expression by Stage and Estrogen Receptor Status in Black and non-Black Patients in CBCS and TCGA. Boxplots displaying continuous log-2 BIRC5 mRNA expression among Black and non-Black breast cancer patients in CBCS (upper panels) and TCGA (lower panels) stratified by (**A**) tumor stage and (**B**) estrogen receptor status. Welsh’s two-sample t-test *p *values listed within each plot. ER: estrogen receptor

The distribution of clinical and demographic characteristics in CBCS reflects its population-based sampling schema, with higher proportions of Black participants, higher proportions of participants < 50 years of age, and higher proportions of ER-negative, Basal-like, and Stage I cases compared to TCGA (Table 1). However, in both TCGA and CBCS, BIRC5-high tumors were more common among Luminal B (LumB), Her2-enriched, Basal-like, ROR-PT-high and ER-negative tumor subtypes, as well as higher-stage tumors, and were more frequent among cases from Black women and younger women (< 50 years of age). BIRC5 is one of the genes used in the PAM50 subtype predictor, so we also performed a sensitivity analysis excluding BIRC5 from the algorithm and found that the distribution of BIRC5-high tumors remained very similar across PAM50 subtypes (Additional file 1: Table S1). Figure 1 shows that in univariate analyses among both CBCS and TCGA, continuous BIRC5 expression differs by race, even after stratification by tumor stage (I, II, III/IV; Fig. 1A) and ER status (positive or negative; Fig. 1B).

**Table 1:** Characteristics of the study population

	CBCS			TCGA		
	Total	BIRC5-Low	BIRC5-High	Total	BIRC5-Low	BIRC5-High
	(n = 2174)	(n = 1630)	(n = 544)	(n = 1095)	(n = 820)	(n = 274)
	n(%)	n(%)	n(%)	n(%)	n(%)	n(%)
Age						
< 50 years	1137 (52.3)	787 (48.3)	350 (64.3)	295 (27)	203 (24.8)	92 (33.6)
≥ 50 years	1037 (47.7)	843 (51.7)	194 (35.7)	798 (73)	616 (75.2)	182 (66.4)
Missing				2	2	
Race						
Non-Black	1061 (48.8)	860 (52.8)	201 (36.9)	816 (81.7)	647 (86.7)	169 (66.8)
Black	1113 (51.2)	770 (47.2)	343 (63.1)	183 (18.3)	99 (13.3)	84 (33.2)
Missing				96	75	21
ER status						
Positive	1389 (64.2)	1196 (73.7)	193 (35.7)	807 (77.2)	683 (87.2)	124 (47.1)
Negative	774 (35.8)	427 (26.3)	347 (64.3)	239 (22.8)	100 (12.8)	139 (52.9)
Missing	11	7	4	49	38	11
PR status						
Positive	1159 (53.8)	1003 (62)	156 (29)	347 (33.2)	607 (77.6)	91 (34.6)
Negative	996 (46.2)	614 (38)	382 (71)	698 (66.8)	175 (22.4)	172 (65.4)
Missing	19	13	6	50	39	11
Her2 status						
Positive	329 (15.2)	244 (15.1)	85 (15.8)	164 (17.9)	127 (18.5)	37 (16.1)
Negative	1829 (84.8)	1375 (84.9)	454 (84.2)	752 (82.1)	559 (81.5)	193 (83.9)
Missing	16	11	5	179	135	44
PAM50 subtype						
LumA	986 (45.4)	954 (58.5)	32 (5.9)	565 (51.7)	540 (65.9)	25 (9.1)
LumB	330 (15.2)	206 (12.6)	124 (22.8)	216 (19.8)	137 (16.7)	79 (28.8)
HER2	195 (9.0)	130 (8.0)	65 (11.9)	82 (7.5)	55 (6.7)	27 (9.9)
Basal	583 (26.8)	263 (16.1)	320 (58.8)	190 (17.4)	50 (6.1)	140 (51.1)
Normal	80 (3.7)	77 (4.7)	3 (0.6)	40 (3.7)	37 (4.5)	3 (1.1)
Tumor stage						
Stage I	722 (33.6)	605 (37.6)	117 (21.8)	182 (17)	154 (19.3)	28 (10.4)
Stage II	1056 (49.2)	741 (46)	315 (58.7)	619 (57.9)	446 (55.8)	173 (64.1)
Stage III	299 (13.9)	214 (13.3)	85 (15.8)	249 (23.3)	186 (23.3)	63 (23.3)
Stage IV	71 (3.3)	51 (3.2)	20 (3.7)	20 (1.9)	14 (1.8)	6 (2.2)
Missing	26	19	7	25	21	4
Tumor size						
≤ 2 cm	978 (45.6)	824 (51.2)	154 (28.7)	NA	NA	NA
2–5 cm	924 (43.1)	629 (39.1)	295 (54.9)	NA	NA	NA
> 5 cm	244 (11.4)	156 (9.7)	88 (16.4)	NA	NA	NA
Missing	28	21	7			
ROR-PT						
Low	532 (24.8)	527 (32.8)	5 (0.9)	237 (22.3)	236 (29.6)	1 (0.4)
Medium	1079 (50.3)	906 (56.3)	173 (32.2)	587 (55.1)	493 (61.9)	94 (35.1)
High	535 (24.9)	176 (10.9)	359 (66.9)	241 (22.6)	68 (8.5)	173 (64.6)
Missing	28	21	7	30	24	6

TCGA: the Cancer genome atlas; CBCS: Carolina breast cancer study; LumA: Luminal A; LumB: Luminal B; HER2: Her2-Enriched; Basal: Basal-like; ER: Estrogen Receptor; NA: not available; ROR-PT: PAM50 Risk of Recurrence Score; Missing values not included in percentages

**Fig. 2:**
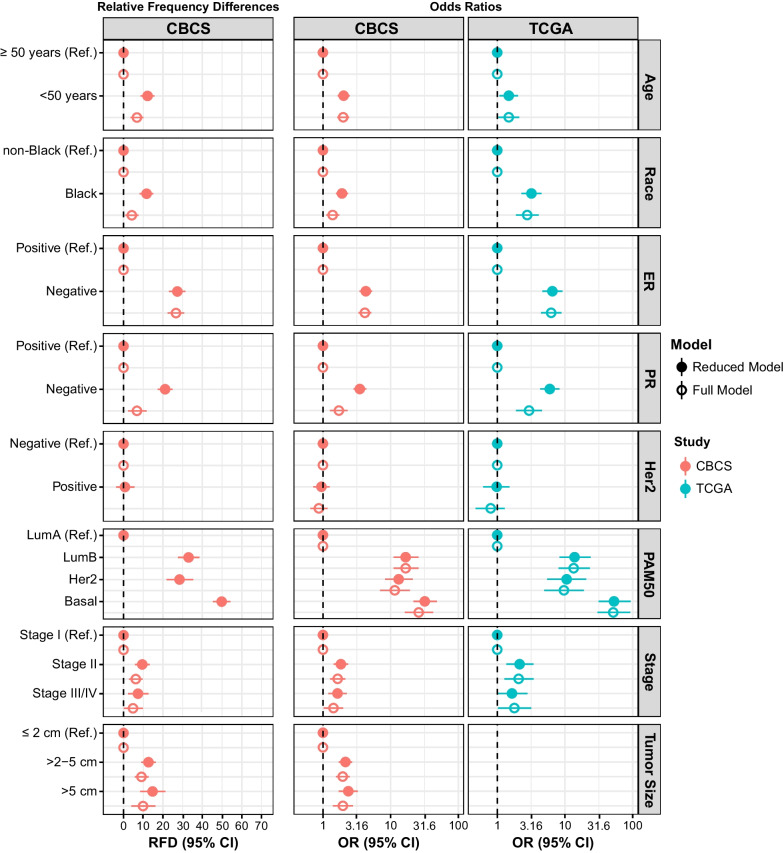
Association between BIRC5-high, patient and tumor characteristics in CBCS and TCGA. Forest plot displaying relative frequency differences and 95% confidence intervals (left panel) and odds ratios (center and right panels) for patient age, race, estrogen receptor/progesterone receptor/Her2 receptor status, PAM50 subtype, stage and tumor size across BIRC5 expression categories in CBCS (left and center panels, red circles) and TCGA (right panel, blue circles). Reduced models were adjusted for age and race according to the CBCS randomized sampling design (solid circles), and additionally adjusted for tumor stage and estrogen receptor status in full models (open circles). RFD: relative frequency difference; OR: odds ratio; 95% CI: 95% confidence interval; ER: estrogen receptor; PR: estrogen receptor; Ref.: Referent; BIRC5 referent group = BIRC5-low for all models. Dashed line represents the null value for each model

We next evaluated associations between categories of BIRC5 expression (i.e., tumors classified as BIRC5-high vs. BIRC5-low, defined as the upper quartile of RNA expression vs. all other quartiles) across the full CBCS and TCGA study populations. In both CBCS and TCGA, similar associations were observed for age at diagnosis, race, ER/PR/HER2 status, PAM50 subtype, tumor stage, and tumor size (Fig. 2, Table 2). To characterize these associations, we estimated relative frequency differences, defined as the difference between the proportions of participants with BIRC5-high tumors in each index group compared to the referent category. In the CBCS, BIRC5-high tumors were 12.1% more frequent among younger participants (< 50 years of age) and 11.7% more frequent among tumors from Black participants. In addition, BIRC5-high displayed strong relationships with aggressive tumor characteristics, with higher frequency among hormone receptor (HR)-negative tumors (BIRC5-high RFD for ER-negative: 27.3%, PR-negative: 21.1%) and aggressive PAM50 subtypes (BIRC5-high RFD for LumB: 33.0%, HER2-Enriched: 28.4%, and Basal-like 49.8%). After additional adjustment for tumor characteristics (e.g., ER status and tumor stage), BIRC5-high remained significantly associated with young age, Black race, ER status, and tumor size (Fig. 2, left panel; Table 2). We also observed that stage II tumors had a higher frequency of BIRC5-high (compared to Stage I), although similar associations with Stage III/IV tumors were attenuated after additional adjustment. We performed a sensitivity analysis excluding non-Black participants that did not self-report White race [N = 56 (2.6%)] and the magnitude of associations in Table 2 were unchanged.

**Table 2:** Associations between BIRC5-High, clinical, and demographic variables in the Carolina breast cancer study and cancer genome atlas

	CBCS		CBCS		TCGA	
	RFD (95% CI)^a^	RFD (95% CI)^b^	OR (95% CI)^a^	OR (95% CI)^b^	OR (95% CI)^a^	OR (95% CI)^b^
Age						
≥ 50 years	Ref	Ref	Ref	Ref	Ref	Ref
< 50 years	12.1 (8.5—15.6)	6.8 (3.6—7.9)	2.0 (1.7—2.5)	2.0 (1.6—2.4)	1.5 (1.1—2.0)	1.5 (1.0—2.1)
Race						
Non-Black	Ref	Ref	Ref	Ref	Ref	Ref
Black	11.7 (8.2—15.1)	4.2 (1.0—7.6)	1.9 (1.6—2.4)	1.4 (1.1—1.7)	3.2 (2.3—4.5)	2.8 (1.9—4.1)
ER status						
ER-Positive	Ref	Ref	Ref	Ref	Ref	Ref
ER-Negative	27.3 (23.1—31.4)	26.6 (22.4—30.8)	4.3 (3.5—5.3)	4.2 (3.4—5.2)	6.5 (4.7—9.2)	6.3 (4.4—8.9)
PR status						
ER-positive	Ref	Ref	Ref	Ref	Ref	Ref
ER-negative	21.1 (17.4—24.9)	6.8 (2.3—11.7)	3.5 (2.8—4.4)	1.7 (1.3—2.3)	6 (4.3—8.3)	3 (1.9—4.6)
Her2 status						
Her2-Negative	Ref	Ref	Ref	Ref	Ref	Ref
Her2-Positive	0.71 (-3.75—5.54)	DNC	1.0 (0.7—1.3)	0.9 (0.7—1.2)	1 (0.6—1.5)	0.8 (0.5—1.3)
PAM50 subtype						
LumA	Ref	Ref	Ref	Ref	Ref	Ref
LumB	33.0 (27.6—38.5)	DNC	16.7 (11.1—25.7)	16.7 (11.1—25.7)	13.9 (8.3—23.9)	13.5 (8.1—23.2)
HER2-enriched	28.4 (21.9—35.3)	DNC	13.1 (8.3—21.2)	11.5 (7.0—19.2)	10.6 (5.5—20.6)	9.7 (4.9—19.0)
Basal	49.8 (45.4—54.2)	DNC	31.9 (21.8—48.2)	26 (16.3—42.5)	53 (31.4—93.2)	51.7 (30.3—91.7)
Tumor stage						
Stage I	Ref	Ref	Ref	Ref	Ref	Ref
Stage II	9.5 (5.8—13.2)	6.2 (2.8—9.7)	1.8 (1.4—2.4)	1.7 (1.3—2.1)	2.1 (1.4—3.4)	2.1 (1.3—3.5)
Stage III/IV	7.3 (2.3—12.7)	4.8 (0.2—9.8)	1.6 (1.2—2.2)	1.4 (1.0—2.0)	1.7 (1.0—2.8)	1.8 (1.0—3.2)
Tumor size						
≤ 2 cm	Ref	Ref	Ref	Ref	NA	NA
> 2–5 cm	12.7 (9.0—16.4)	9.1 (5.7—12.7)	2.2 (1.7—2.7)	2 (1.6—2.5)	NA	NA
> 5 cm	14.7 (8.5—21.3)	9.9 (4.0—16.2)	2.4 (1.7—3.3)	2 (1.4—2.8)	NA	NA

TCGA: The Cancer Genome Atlas; CBCS: Carolina Breast Cancer Study; Lum A: Luminal A; LumB: Luminal B; ER: Estrogen Receptor; 95% CI: 95% confidence interval; DNC: does not converge; NA: Not available; RFD: relative frequency difference; OR: Odds Ratio. Ref.: Referent; Referent group = BIRC5-Low for all models. Null value for RFD models = 0.0; Null value for OR models = 1.0

^a^Models adjusted for race and age. ^b^ Models adjusted for race, age, tumor stage and ER status

We also present odds ratios from multivariate logistic regression models, which converge better with the smaller cell sizes present in TCGA. Figure 2 displays odds ratios for the association between BIRC5-high, patient, and tumor characteristics in CBCS (center panel) and TCGA (right panel), which mirrored relationships observed across CBCS. In both studies, younger participants and Black participants had higher odds of BIRC5-high, as did tumors with HR-negative status, aggressive PAM50 subtypes, advanced stage, and larger size. These relationships, including associations between young age and Black race, remained significant after additional adjustment for tumor characteristics, but associations with Stage III/IV were attenuated. No relationship was observed between BIRC5 and clinical Her2 status in either CBCS or TCGA, which may be due to the low proportion of Her2-positive cases in each dataset (Her2-positive cases: CBCS n = 329; TCGA n = 164). Thus, BIRC5 is highly correlated with aggressive tumor features. Further, age and race seem to be important factors contributing to BIRC5 levels, even after adjusting for tumor characteristics.

### Prognostic utility of BIRC5

**Fig. 3:**
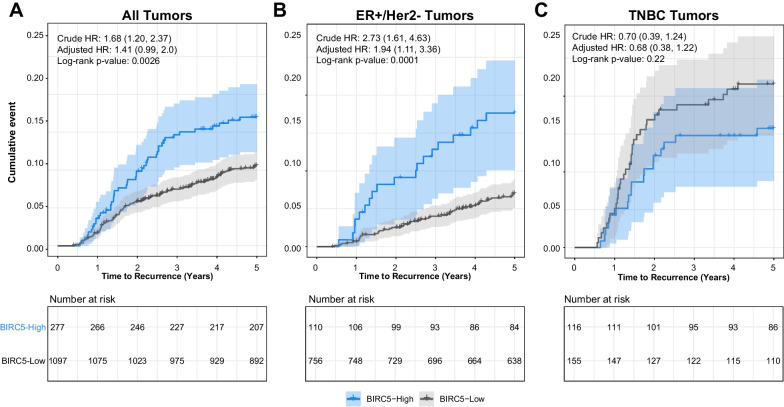
Five-year recurrence-free survival (RFS) by BIRC5 expression status in CBCS. Kaplan–Meier survival analysis illustrating 5 year RFS in (**A**) all CBCS phase 3 cases, (**B**) among ER-positive/Her2-negative tumors only and (**C**) among triple-negative tumors only. Cox proportional hazard ratios and 95% confidence intervals adjusted for patient age and tumor stage are displayed within each plot for BIRC5-high relative to BIRC5-low tumors. All analyses were restricted to stage I-III tumors. Tick marks represent censored individuals. Shaded regions represent 95% confidence intervals for each group. ER: estrogen receptor; TNBC: triple-negative breast cancer; HR: hazard ratio; 95% CI: 95% Confidence Interval. Referent group = BIRC5-low for CoxPH models.

We hypothesized that BIRC5 is associated with early (5-year) recurrence. The CBCS Phase 3 identified 159 recurrences during the first 5 years of follow-up. Among all tumors, BIRC5-high tumors had higher recurrence in univariate models, but not in multivariate models [Crude HR (95% CI): 1.68 (1.20, 2.37); Adjusted HR (95% CI): 1.41 (0.99, 2.0)] (Fig. 3A). However, in stratified analyses, BIRC5-high was significantly associated with recurrence only among ER-positive/Her2-negative tumors [Crude HR (95% CI): 2.73 (1.61, 4.63); Adjusted HR (95% CI): 1.94 (1.11, 3.36)] (Fig. 3B). No significant associations with recurrence were observed among TNBC cases [Crude HR (95% CI): 0.7 (0.39, 1.24); Adjusted HR (95% CI): 0.68 (0.38, 1.22)] (Fig. 3C). In a sensitivity analysis, we additionally adjusted recurrence models for race and found that BIRC5-High remained significantly associated with recurrence only among ER-positive/Her2-negative tumors, although hazard ratios were slightly attenuated [Adjusted HR (95% CI), All tumors: 1.34 (0.94, 1.91); ER + /Her2-: 1.91 (1.10, 3.32); TNBC: 0.67 (0.37, 1.21)]. However, adjusting for race, herein considered a social construct, is difficult to interpret due to differential distribution of multiple biological treatment, and health care access factors. We also performed sensitivity analyses restricting to participants that were chemo treated (ER + /HER2-: 54.5%; TNBC: 94.1%) or restricting to those who initiated endocrine therapy (ER + /HER2-: 90.3%), and the magnitude of the HRs were not substantially altered.

## Discussion

In this analysis, BIRC5/survivin was investigated as a biomarker in two large studies representing 3269 patients with breast cancer, including TCGA and the CBCS, a large and diverse population-based study enriched for Black and younger patients. In both studies, BIRC5 was associated with high-risk populations, including participants with aggressive tumor subtypes, advanced stage and larger tumors. Young women and Black women also had higher frequencies of BIRC5-high tumors. These differences persisted after adjustment for ER status and tumor stage, suggesting that BIRC5 associations are not driven exclusively by subtype and stage and may reflect additional biological, genetic or environmental influences. Higher BIRC5 was also prognostic for early recurrence among ER-positive participants in the CBCS, which is important given that the disparities in recurrence between Black and White women are greatest among ER-positive breast cancer [25, 36–40].

The results of our study aligns with prior work demonstrating an association between high survivin expression and aggressive breast tumor features including hormone receptor negativity, higher stage, larger size, and non-Luminal A subtype [20–23, 40], all of which remained significantly associated, independent of estrogen receptor status. BIRC5/survivin expression has also previously been reported as an independent marker of poor prognosis in breast cancer [24, 41] however, the findings of the current analysis extend those prior investigations to a large and diverse patient population. The prognostic relationship with poorer RFS persists in this study. Given our finding of higher BIRC5/survivin and a previous study showing increased survivin phosphorylation in tumors from Black women [42], the burden of BIRC5-high may be particularly important for Black patients.

Our findings also shed light on previously reported BIRC5 associations with breast cancer clinical outcomes [24, 41, 43, 44], which have seldom been stratified by clinical subtype. While BIRC5-high was more prevalent among TNBC tumors, BIRC5 had the strongest prognostic value among ER-positive/Her2-negative disease. This was also seen in the METABRIC cohort presented by Oparina et al., [40] where BIRC5 was only prognostic in the ER + group and not the ER- group. In contrast, Zhang et al [43] showed that survivin predicted survival in 136 TNBC patients. These inconsistent findings across studies highlight that variables mediating BIRC5/survivin responses remain poorly understood. One hypothesis is that in TNBC — a truly distinct disease with its own set of hallmark mutations [35], levels of genomic instability, and underlying tumor immune microenvironment — BIRC5/survivin has a distinct relationship with survival. Elucidating mediating events will be essential to understanding the treatment prospective of anti-survivin therapies. Based on our current results, BIRC5-targeted therapies may be valuable, especially for patients with ER-positive tumors, the subtype with the largest Black-White outcomes disparity [25, 36–40].

There is high feasibility of translating anti-survivin therapy to breast cancer, as it has already been pursued as a cancer therapeutic target by various strategies [27, 45–47], and is already measured in the clinic on the validated prognostic assays, Prosigna [19] and Oncotype DX [18]. A strength of our study was the ability to exclude BIRC5 from the PAM50 algorithm (the research version of the Prosigna assay) to independently assess BIRC5/survivin as a high-risk biomarker in breast cancer and its relationship with tumor subtype. Another strength was the use of a large, diverse population-based cohort that represents the natural distribution of breast cancer in the population, and for which RNA expression profiling was optimized for FFPE specimens. However, our analysis also had limitations. A limitation of our findings is that while we observed differences in BIRC5 expression by race, we are unable to evaluate the differential effects of BIRC5 in context of the social construct of race. Our targeted approach also does not allow for the investigation of survivin splice variants, which have been suggested to differ in function and according to prognostic significance [48, 49]. Further studies investigating the role of different survivin splice variants in diverse populations may be necessary for therapeutic stratification. Another limitation was the low number of HER2-positive tumors in our dataset, which did not allow for assessment of BIRC5-mediated recurrence among HER2-positive cases. Future studies should also consider longer follow-up times and detailed chemotherapy data to further disentangle the relationship between race, age, tumor subtype and survivin. Our results fill a research gap in understanding the potential role of survivin in breast cancer disparities, and possibly provide future insight into treatment strategies for a cohort of women with unmet clinical needs. Further studies are needed to help close this gap which constitutes the largest disparity among cancer-specific diseases.

### Supplementary Information

**Additional file 1. Supplemental Table 1.** Sensitivity analysis assessing the distribution of PAM50 tumor subtypes across BIRC5-high and BIRC5-low tumors in the CBCS, excluding BIRC5 as one of the genes in the PAM50 algorithm

## Data Availability

TCGA RNA-seq data are publicly available under dbGaP accession phs000178.v1.p1, with additional data available at https://gdc.cancer.gov/about-data/publications/PanCan-CellOfOrigin[35]. CBCS data are available upon request (https://unclineberger.org/cbcs).

## Abbreviations

CBCS: Carolina breast cancer study
TCGA: The cancer genome atlas
NC: North Carolina
UNC-CH: University of North Carolina at Chapel Hill
TNBC: Triple-negative breast cancer
ER: Estrogen receptor
IHC: Immunohistochemical
RFS: Recurrence-free survival
FFPE: Formalin-fixed, paraffin-embedded
RUV: Remove unwanted variation
ROR-PT: Risk of recurrence incorporating proliferation and tumor size
glm: Generalized linear models
RFD: Relative frequency difference
95% CI: 95% Confidence interval
